# Supplementary material for: Ultraflat Cu(111) foils by surface acoustic wave-assisted annealing
Source: Nat Commun. 2024 Nov 2;15:9488. doi: 10.1038/s41467-024-53573-y (PMC11531502; doi:10.1038/s41467-024-53573-y)
Supplement: Supplementary file 1 — Supplementary Information [file 41467_2024_53573_MOESM1_ESM.pdf]

Supplementary Information for

Ultraflat Cu(111) Foils by Surface Acoustic Wave Assisted Annealing

Bo Tian[1†*], Junzhu Li[1,2†], Qingxiao Wang[3], Abdus Samad[2], Yue Yuan[2], Mohamed Nejib Hedhili[3], Arun Jangir[2], Marco Gruenewald[4], Mario Lanza[2], Udo Schwingenschlögl[2], Torsten Fritz[4], Xixiang Zhang[2*], Zheng Liu[1*]

[1]School of Materials Science and Engineering, Nanyang Technological University, Singapore 639798, Singapore.

[2]Physical Science and Engineering Division, King Abdullah University of Science and Technology (KAUST), Thuwal 23955-6900, Saudi Arabia.

[3]Imaging and Characterization Core Lab, King Abdullah University of Science and Technology (KAUST), Thuwal, 23955-6900, Saudi Arabia.

[4]Institute of Solid State Physics (IFK), Friedrich Schiller University Jena, Jena 07743, Germany.

[†]These authors contributed equally.

*E-mail: botianlab@gmail.com; xixiang.zhang@kaust.edu.sa; z.liu@ntu.edu.sg

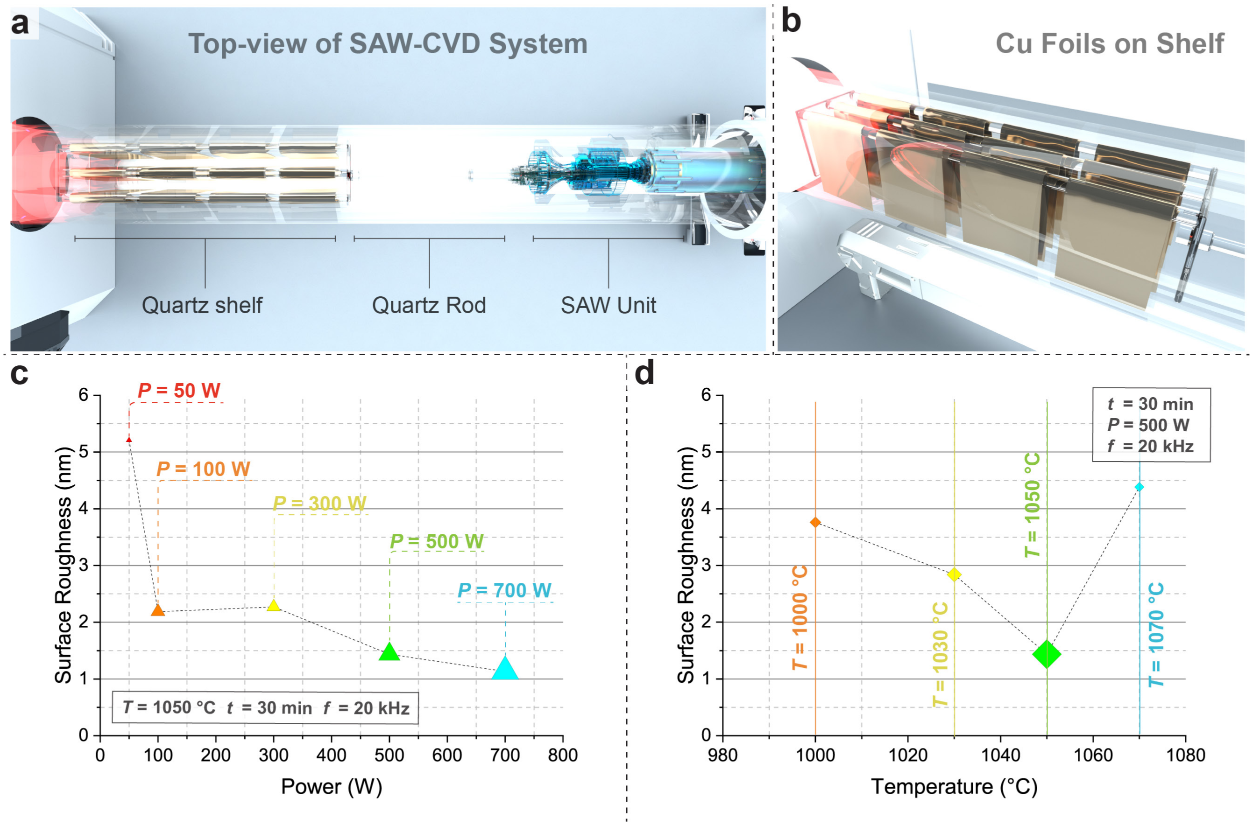

Supplementary Fig. 1 | SAW treatment experiments. a, Top view of the SAW treatment system, which integrates a traditional chemical vapor deposition (CVD) system with a SAW unit connected to a quartz shelf via a quartz rod. The Cu foils are suspended on the quartz shelf and moved into the high-temperature zone for SAW-annealing treatment. **b**, Schematic illustration of the Cu foils positioned on the quartz shelf. **c-d**, Surface roughness measurements of the treated Cu foil under varying SAW power levels (50 W, 100 W, 300 W, 500 W, and 700 W) and different annealing temperatures (1000 °C, 1030 °C, 1050 °C, and 1070 °C). Notably, while higher temperatures typically reduce surface roughness, our experiments showed an increase in roughness at 1070°C. We attribute this to excessive evaporation of Cu surface atoms, caused by the high annealing temperature combined with the additional energy input from the SAW process.

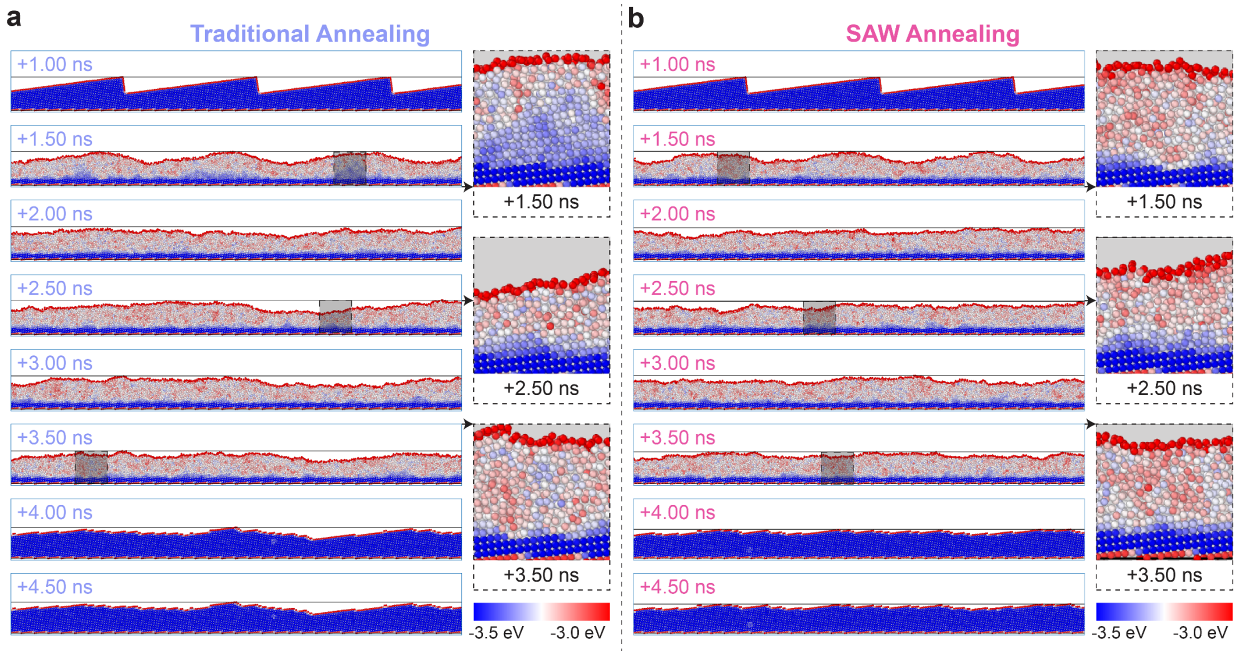

Supplementary Fig. 2 | MD simulations of a Cu surface. a-b, Snapshots from MD simulations at different time points for traditional annealing at 1323 K (a) and SAW-assisted annealing at 1323 K (b). The average potential energy of Cu atoms was calculated and color-coded, ranging from -3.5 eV to -3.0 eV. Detailed atom energy distributions are highlighted for 1.5, 2.5, and 3.5 ns to allow for clearer observations.

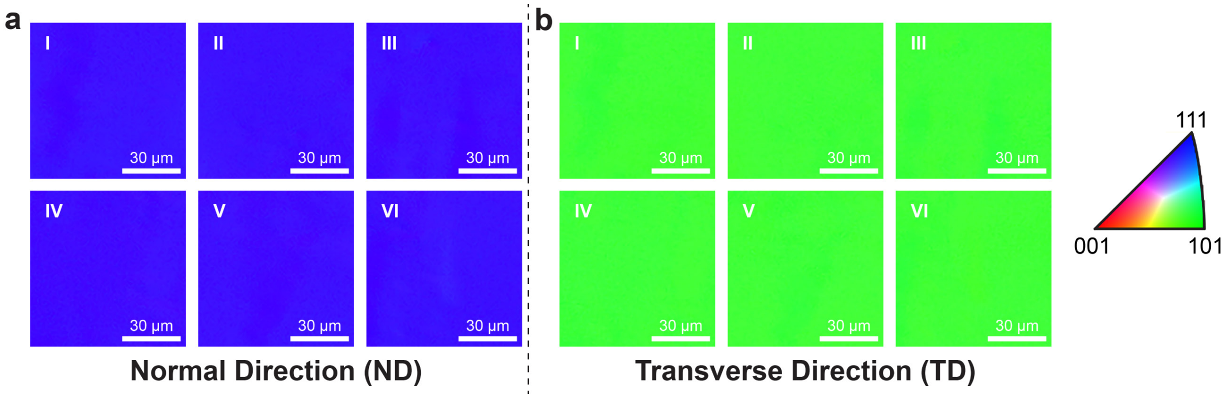

Supplementary Fig. 3 | Electron backscatter diffraction (EBSD) inverse pole figure (IPF) characterization of produced Cu(111) foil. a-b, EBSD IPF maps of the normal (a) and transverse (b) directions collected from six random positions of the Cu (111) foil.

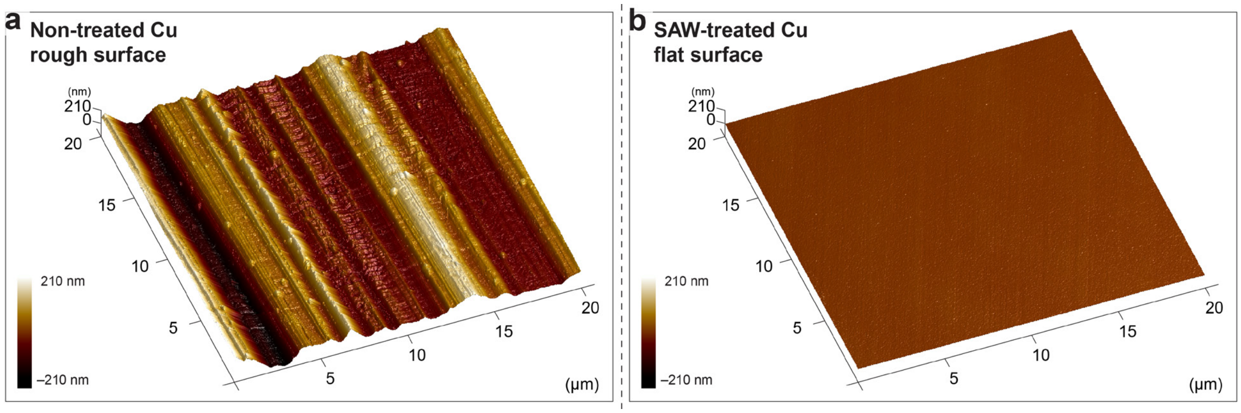

Supplementary Fig. 4 | AFM characterizations. a-b, 3D AFM topography maps of the non-treated Cu foil (a) and SAW-treated Cu foil (b) in the measurement area of 20 × 20 μm^2; The same z-axis range is used for both maps for comparison.

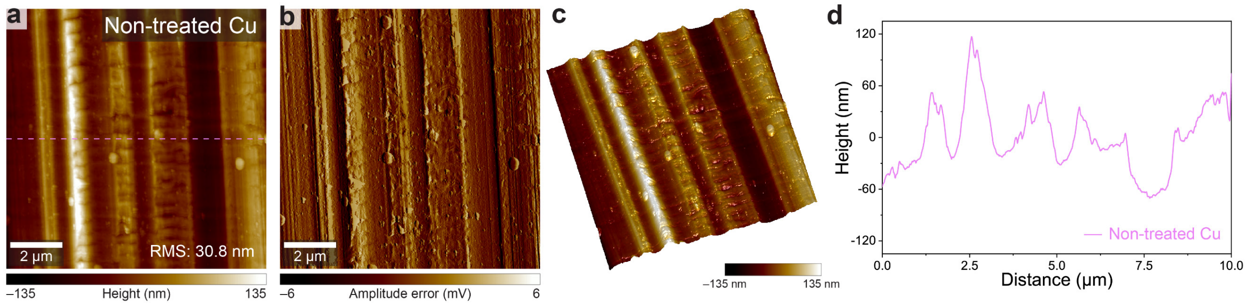

Supplementary Fig. 5 | AFM images of as-received Cu foils. a-c, AFM topography (a), amplitude error (b), and 3D map (c) images of non-treated Cu foil in the measurement area of 10 × 10 μm^2. The surface roughness was calculated as 30.8 nm. **d**, Line profiles for non-treated Cu surface extracted from AFM maps in (a).

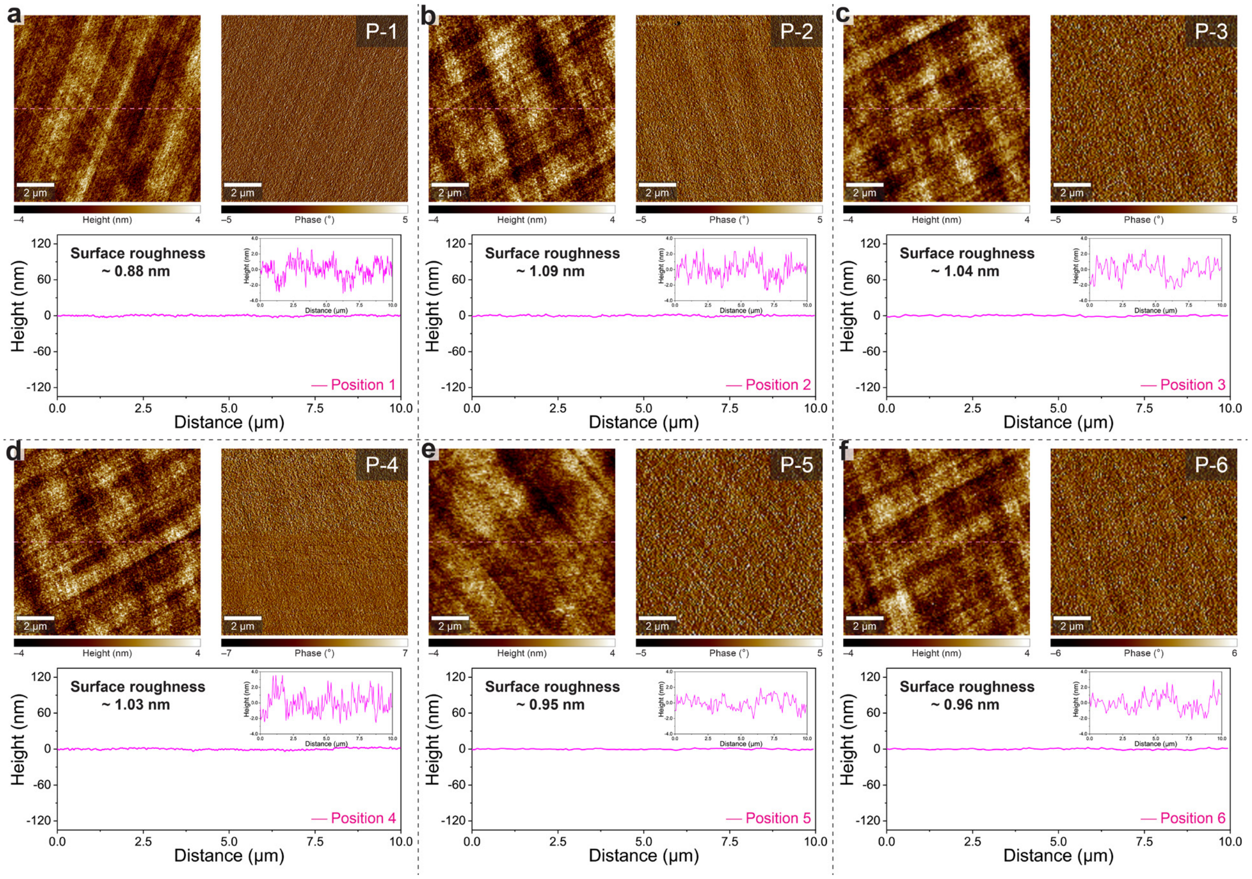

Supplementary Fig. 6 | Surface roughness measurements of SAW-treated Cu(111) foils. a-f, AFM topography (left) and phase (right) maps of SAW-annealed flat Cu foils from six randomly selected locations, with each measurement area covering 10 × 10 μm². The average surface roughness was calculated to be 0.99 nm. Line profiles extracted from each AFM topography map are shown below.

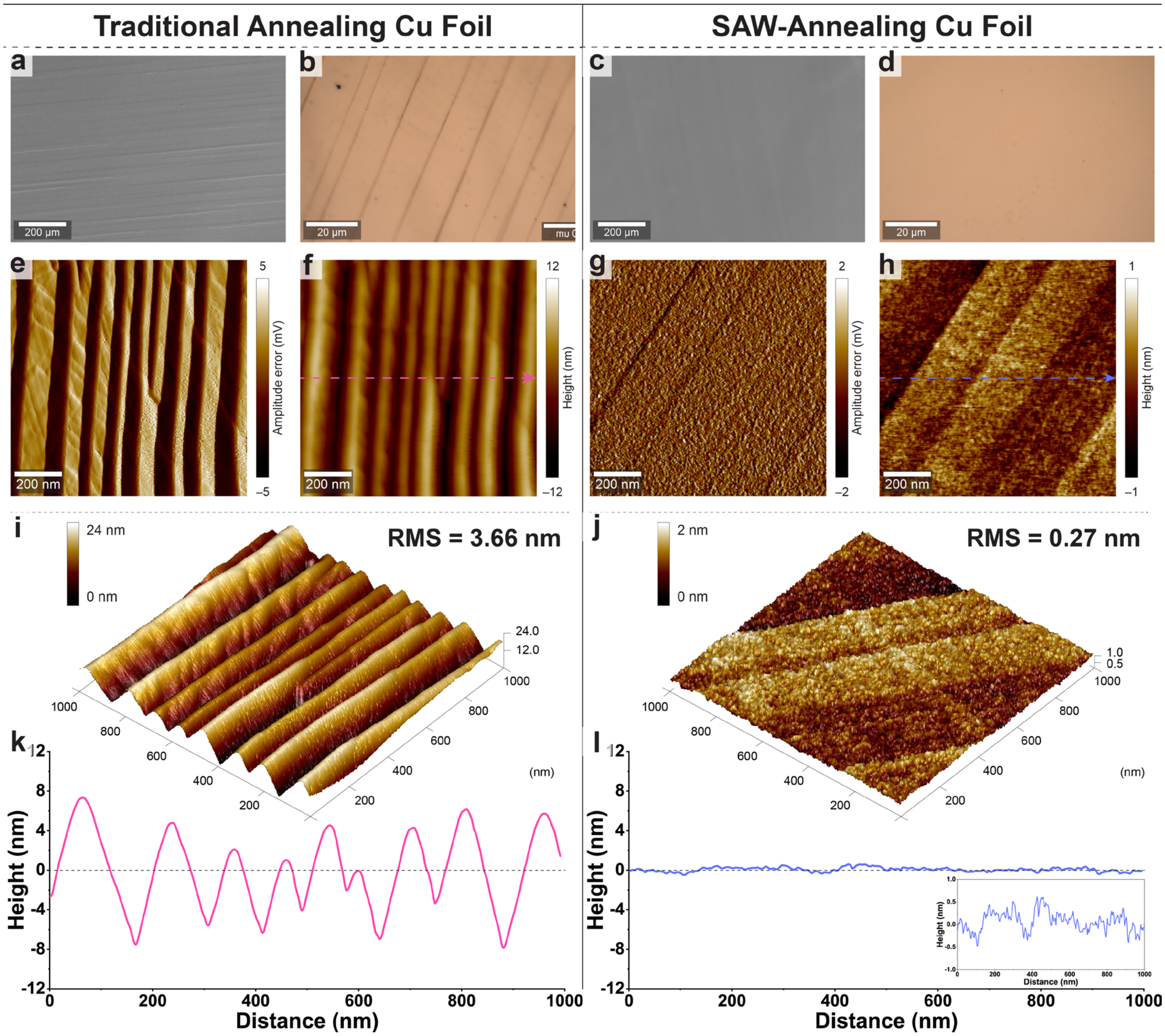

Supplementary Fig. 7 | Comparison of traditional and SAW-assisted annealing of Cu foils. **a-d**, Large-area SEM and optical images of Cu foils annealed using the traditional method (a, b) and the SAW-assisted annealing method (c, d). **e-h**, AFM images of Cu foils subjected to traditional annealing (e, f) and SAW-assisted annealing (g, h). **i-j**, AFM 3D map images of Cu foils after traditional annealing (i) and SAW-assisted annealing (j), with surface roughness measured at 3.66 nm and 0.27 nm, respectively, over a 1.0 × 1.0 μm² area. **k-l**, Line profiles of Cu surfaces from traditional annealing and SAW-assisted annealing, extracted from the AFM maps in (f) and (h).

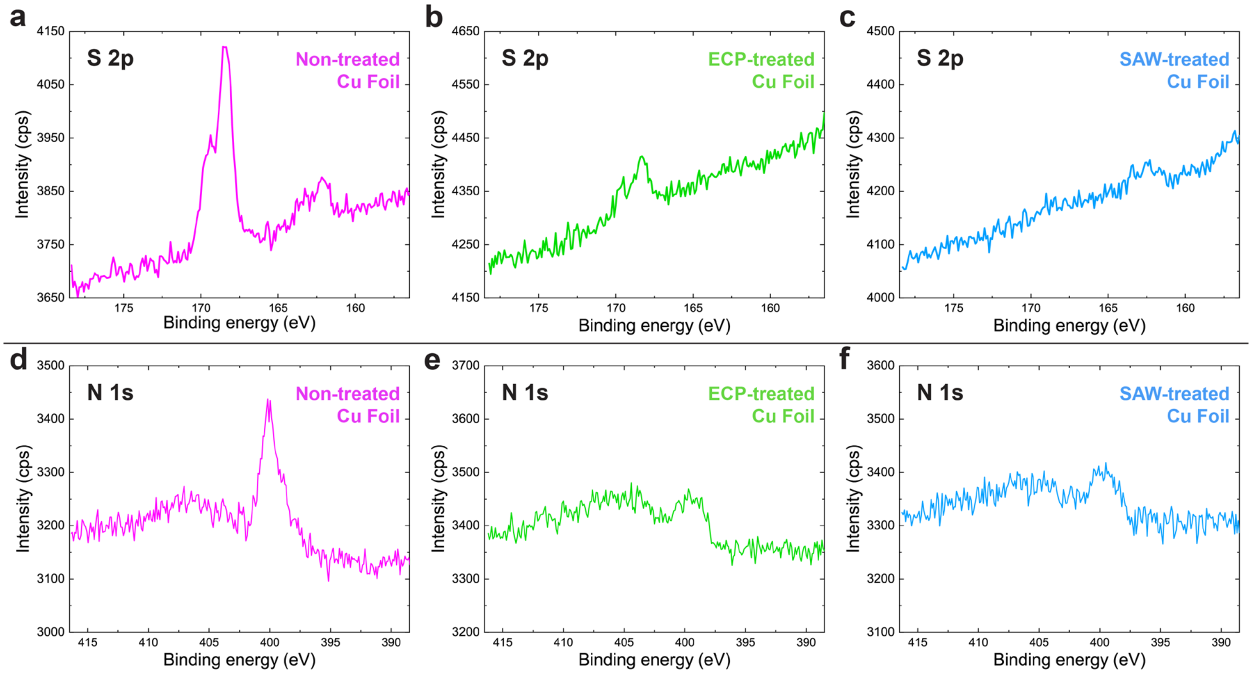

Supplementary Fig. 8 | XPS spectra of potential contaminants in Cu foils. a-c, XPS spectra of S 2p core level collected from the non-treated (a), ECP-treated (b), and SAW-treated (c) Cu foils. **d-f**, XPS spectra of N 1s core level collected from the non-treated (d), ECP-treated (e), and SAW-treated (f) Cu foils.

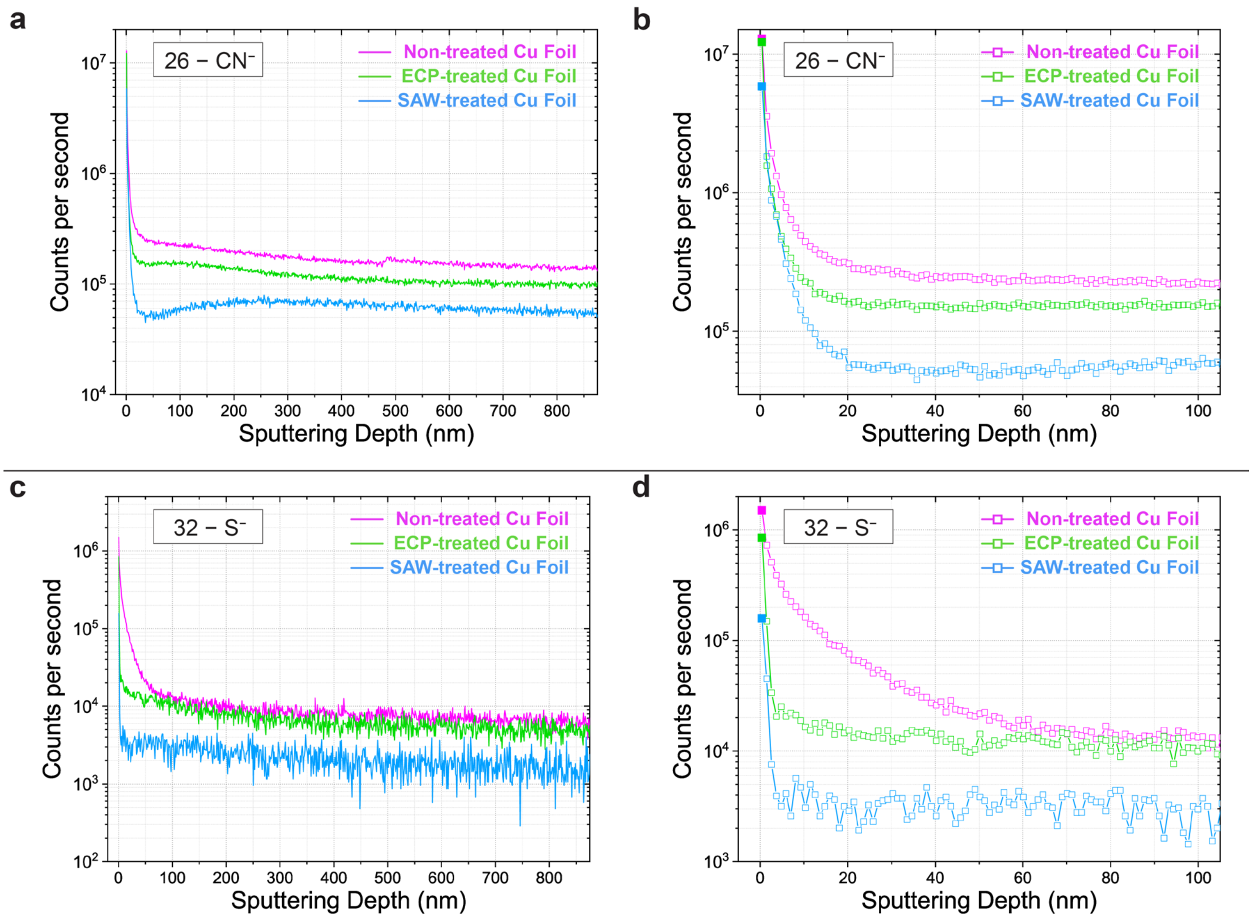

Supplementary Fig. 9 | SIMS measurements of Cu foils. a-b, D-SIMS spectra of nitrogen contaminate intensity inside three types of Cu foils. **c-d**, D-SIMS spectra of sulfur contaminate intensity inside three types of Cu foils.

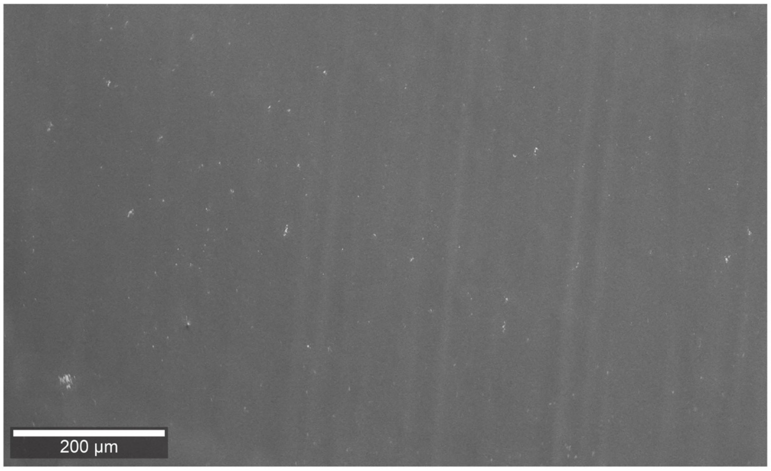

Supplementary Fig. 10 | Large-scale SEM image of as-grown monolayer graphene on Cu(111) foil.

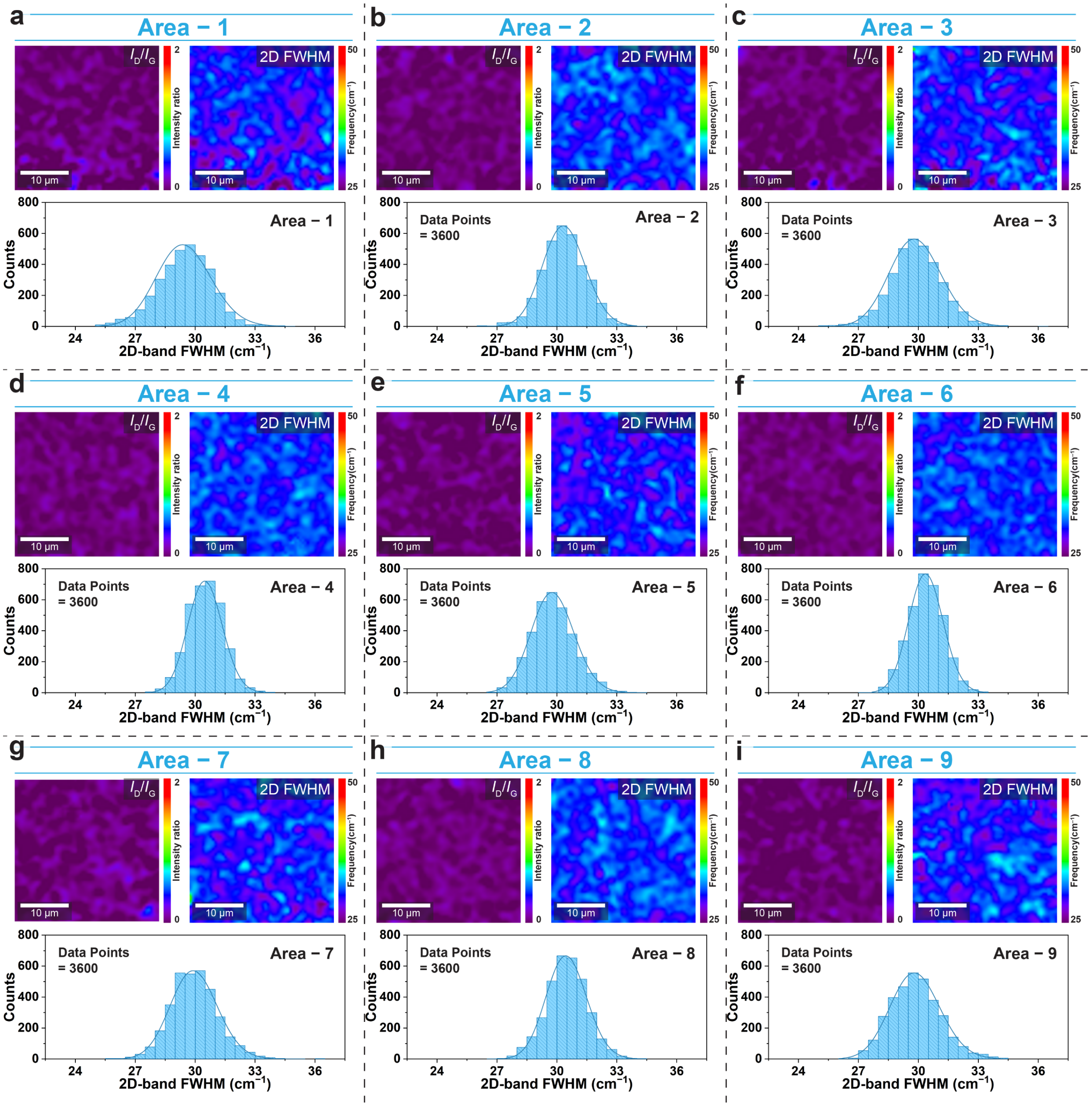

Supplementary Fig. 11 | Raman maps of graphene grown on flat Cu(111) foil and transferred onto a SiO_2/Si substrate. a-i, I_D/I_G ratio and 2D FWHM Raman maps collected from nine randomly selected locations from the graphene film on the SiO_2/Si wafer. The statistical FWHM values extracted from each map are presented in the lower panel.

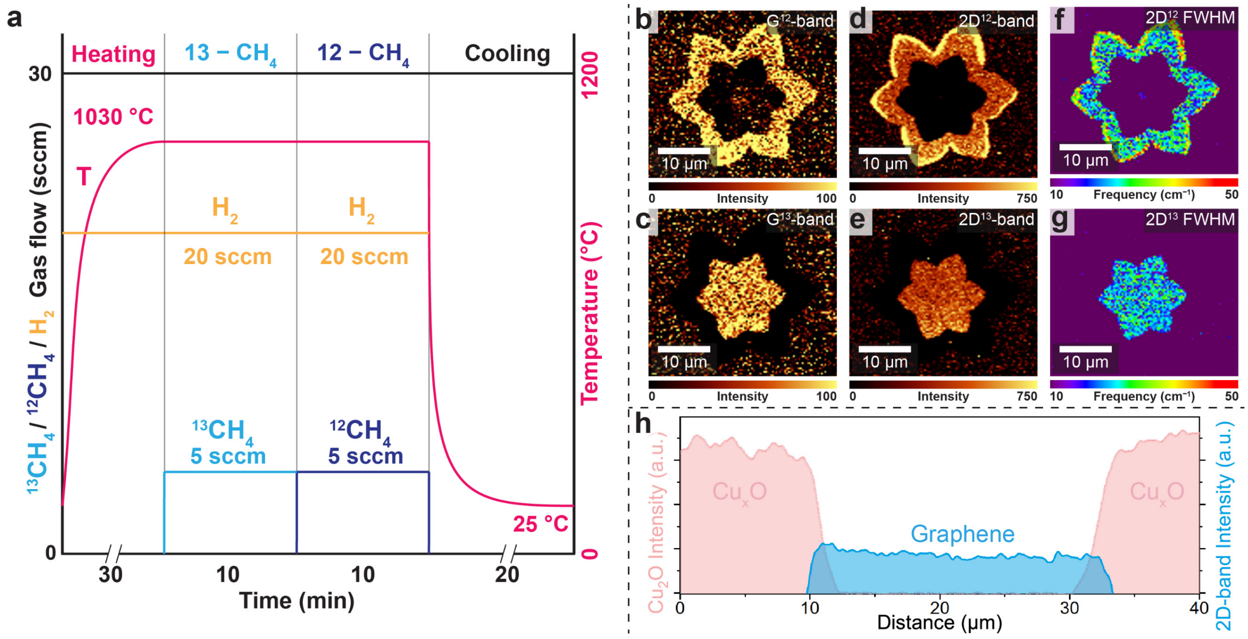

Supplementary Fig. 12 | Graphene islands grown on Cu foil after natural oxidation. a, Schematic diagram of graphene island growth on flat Cu foil in a CVD system, showing the growth parameters, including gas flow ratios of ^{13}C-CH$_4$, ^{12}C-CH$_4$, H_2, and growth temperature during the CVD growth process. **b-c**, Raman maps of the ^{12}C-G (b) and ^{13}C-G (c) band intensities. **d-e**, Raman maps of the ^{12}C-2D (d) and ^{13}C-2D (e) band intensities. **f-g**, Raman maps of 2D FWHM of ^{12}C (f) and ^{13}C (g) graphene regions. **h**, Raman intensity line profiles of Cu_xO and graphene-2D bands along the dashed line in Fig. 3g.

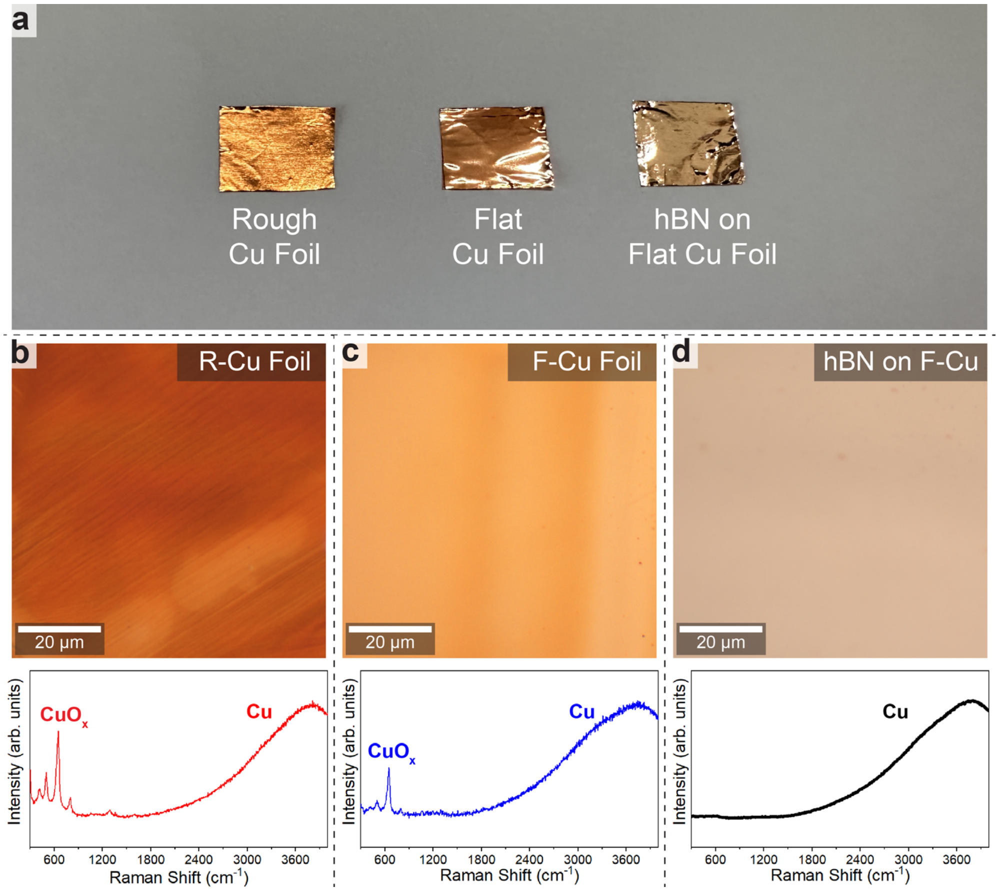

Supplementary Fig. 13 | Oxidation experiments on rough and flat Cu foils. a, Photograph of rough Cu foil, SAW-treated flat Cu foil, and flat Cu foils covered with a monolayer of hBN after undergoing oxidation treatment. **b-d**, Optical images acquired from rough Cu foil, SAW-treated flat Cu foil, and flat Cu foils with monolayer hBN coverage. Typical Raman spectra for each of these samples are displayed below.
